# Migraine and Tension-Type Headache Are Associated with Multiple Sclerosis: A Case–Control Study

**DOI:** 10.3390/jcm14082778

**Published:** 2025-04-17

**Authors:** Panagiotis Gklinos, Maria-Eleftheria Evangelopoulos, Georgios Velonakis, Dimos Dimitrios Mitsikostas

**Affiliations:** 1 First Neurology Department, Eginition University Hospital, Medical School, National and Kapodistrian University of Athens, 11528 Athens, Greece; 2 Research Unit of Radiology and Medical Imaging, National and Kapodistrian University of Athens, 11528 Athens, Greece; 3 Department of Radiology, General University Hospital “Attikon”, National and Kapodistrian University of Athens, 12462 Athens, Greece

**Keywords:** multiple sclerosis, headache, migraine, tension-type headache, magnetic resonance imaging, case–control study

## Abstract

**Background/Objectives**: Over the past few decades, there has been increased scientific interest in the prevalence of headache disorders among people with MS (pwMS). Although the latest data suggest an association between migraine and multiple sclerosis, studies have been providing inconsistent results largely due to methodological differences, including small sample sizes, lack of control groups, absence of structured headache diaries, and variability in diagnostic criteria. This study aims to address the question of whether pwMS have a higher prevalence of primary headache disorders than healthy controls (HCs) and whether MS is associated with an increased risk of headaches. **Methods**: In this cross-sectional, case–control study, consecutive pwMS from Eginition University Hospital, Athens, Greece, along with matched HCs, were recruited. Both groups were assessed for headache disorders, over the past 3 months from the day of recruitment, using a semi-structured questionnaire and diagnosed according to the International Classification for Headache Disorders 3 (ICHD-3) criteria. A multivariable logistic regression model adjusted for age and sex evaluated the association between MS and headache disorders. **Results**: Ninety-six pwMS and ninety-six matched HCs met the inclusion criteria and were enrolled in the study. A higher prevalence of primary headache disorders in pwMS (71.9%) compared to HCs (43.8%) was observed. Specifically, 28.1% of pwMS had migraine, and 38.5% had tension-type headache (TTH). PwMS were significantly more likely to be diagnosed with any primary headache disorder (OR = 4.54; 95% CI: 2.28 to 9.04; *p* = 1.7), migraine (OR = 2.21 95% CI: 1.05 to 4.62; *p* < 0.05), and TTH (OR = 2.16 95% CI: 1.16 to 4; *p* < 0.05) compared to HCs. **Conclusions**: Our study suggests that primary headache disorders are more prevalent in pwMS in a cohort recruited from the MS outpatient clinic at Eginition University Hospital in Athens, Greece, compared to the general population and highlights the need for targeted headache management within this group. Prospective longitudinal studies are needed to draw more robust conclusions on a potential association and its underlying mechanisms.

## 1. Introduction

Multiple sclerosis (MS) and migraine share similar demographic and epidemiological features. Both conditions predominantly affect women and typically present in young adulthood [1,2]. Moreover, they are more prevalent in Caucasian populations compared to Asian and African ones. These similarities have sparked significant scientific interest in a possible link between the two conditions [3,4]. Several cross-sectional and case–control studies have explored the prevalence of headache disorders and a potential association between headache and MS, but the results have been inconsistent [5,6,7,8,9,10,11,12,13,14,15,16,17,18,19,20,21,22,23,24,25,26,27]. These discrepancies may stem from methodological limitations such as the absence of structured headache diaries, small sample sizes, the lack of control groups, and retrospective study designs. In the past decade, two meta-analyses have been published examining the potential association between migraine and MS. These studies report that people with MS are about twice as likely to be diagnosed with migraine, while tension-type headache (TTH) has not been shown to be associated with MS [28,29].

The association between MS and migraine extends beyond shared demographic characteristics. Emerging evidence suggests that MS-related brain lesions may contribute to headache susceptibility through altered pain modulation and central sensitization mechanisms [30,31]. Additionally, MS-associated immune dysregulation, including pro-inflammatory cytokine activity, lymphocyte and microglial activation, may further exacerbate the pathogenetic mechanisms of migraine [32,33]. This process is possibly mediated through the elevation of calcitonin gene-related peptide (CGRP) levels, a molecule which has been shown to be heavily involved in the pathogenesis of migraine over the past few years [32]. Among the theories that have been proposed to explain the potential link, the most prominent involves the presence of lesions in brain areas involved in pain modulation and perception [30,31]. These areas include cortical regions, the thalamus, and brainstem nuclei such as the periaqueductal gray (PAG), the dorsal raphe nucleus (DRN) in the midbrain, and the locus coeruleus (LC) in the pons. The thalamus is significant to the pathophysiology of primary headache disorders, serving as a relay center for pain signals. It modulates pain perception and processes sensory information. Alterations in thalamic activity are believed to enhance pain sensitivity, leading to increased pain. The PAG is heavily involved in descending pain modulation, particularly through the inhibition of central sensitization at the level of the second- and third-order trigeminovascular neurons [30]. Its activation has been found to mitigate the transmission of pain signals to the brainstem. The DRN is the largest serotonergic nucleus in the brain and plays a role in the modulation of pain through its descending projections that can inhibit pain signals [31]. The LC, on the other hand, plays a pivotal role in regulating arousal, attention, and pain through noradrenergic modulation [31]. The presence of demyelinating lesions within these brain areas that are involved in pain transmission and modulation could trigger the pathophysiological cascades of migraine through amplified pain signal transmission and the perception or dysregulation of descending pain modulation at the brainstem level, leading to the reduced inhibition of pain transmission and increased central sensitization.

Another hypothesis considers the immunological changes in people with MS, particularly in activated lymphocyte populations. Notably, lymphoid follicle-like B-cell structures within the meninges, which represent one of the main drivers of progressive MS through compartmentalized inflammation, are emerging as promising treatment targets. Recent studies indicate that these activated lymphocytes express elevated levels of CGRP, a key player in migraine pathogenesis [32,33]. Consequently, MS patients might face a higher risk of migraines due to increased CGRP levels. Additionally, mast cells in the dura mater, influenced by CGRP and substance-P, release histamine, serotonin, heparin, tryptase, and various cytokines such as IL-1β and IL-6. This release contributes to increased neurogenic inflammation typical of migraine and directly sensitizes trigeminal afferents, leading to prolonged central sensitization [32,33].

**Figure 1:**
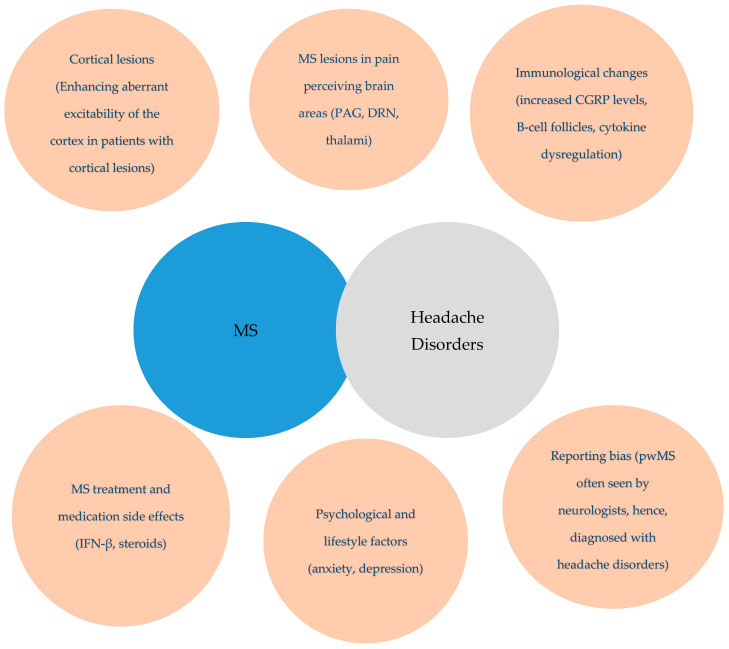
Theories attempting to explain the increased prevalence of headache disorders in pwMS. MS: multiple sclerosis; PwMS: people with multiple sclerosis; PAG: periaqueductal gray; DRN: dorsal raphe nucleus; CGRP: calcitonin gene-related peptide; IFN-β: interferon beta.

Finally, the increased prevalence of headaches in pwMS may, in part, reflect reporting bias, as pwMS are more frequently seen by neurologists, a fact that could lead to a higher likelihood of headache diagnosis. Additionally, specific MS treatments, including interferon-beta and corticosteroids, are known to trigger or exacerbate headaches, while psychiatric comorbidities such as anxiety and depression, which are more commonly encountered by pwMS, may contribute to the increased prevalence of headache in pwMS [34,35,36]. The proposed theories explaining the increased occurrence of headache disorders in pwMS are summarized in Figure 1.

The primary objective of this study is to investigate the prevalence of primary headache disorders in pwMS in a cohort recruited from the MS outpatient clinic at Eginition University Hospital in Athens, Greece, compared to HCs and to explore whether headache disorders, including migraine and TTH, are more common in pwMS. Given the inconsistent findings of previous studies, our research aims to provide more robust evidence by using a well-defined case–control design with standardized diagnostic criteria and semi-structured headache diaries. By examining these, we strive to improve the understanding of headache disorders in MS and their potential clinical implications for patient management. Based on the previous literature, we hypothesized that pwMS would exhibit a higher prevalence of primary headache disorders, including migraine and tension-type headache, compared to age- and sex-matched healthy controls.

## 2. Materials and Methods

### 2.1. Study Design and Participants

The study used a case–control design to compare the prevalence of primary headache disorders between pwMS and HCs and was conducted and reported in accordance with the STROBE (Strengthening the Reporting of Observational Studies in Epidemiology) guidelines. A completed checklist is included in the Supplementary Material. A retrospective headache assessment over the past three months from the day of recruitment was chosen to provide a practical yet reliable evaluation of headache prevalence. Prior to conducting the study, a power calculation was performed to determine the adequacy of the sample size. Based on data published from the World Health Organization (WHO) and from recent cross-sectional studies, we assumed an effect size of 0.22—the difference in headache prevalence between MS patients (62%) and the general population (40%) [26,37]. With a significance level set at 0.05 and a power of 80%, the power calculation suggested that a total of 178 participants (89 pwMS and 89 healthy controls) would be required to detect statistically significant differences between the groups. This power analysis was conducted using the OpenEpi Version 3 calculator. Consecutive MS patients from the MS outpatient clinic at Eginition University Hospital in Athens, Greece, and age- and sex- matched HC were enrolled between October 2022 and March 2023.

### 2.2. Inclusion and Exclusion Criteria

Inclusion and exclusion criteria were carefully selected to ensure a well-defined study population while minimizing confounding factors, such as secondary headache disorders, recent relapses, and corticosteroid use. Inclusion criteria required MS patients to meet the 2017 revised McDonald criteria and be 18 years or older [38]. HCs were matched to the MS group based on age and sex and were included if they had no history of neurological disorders. Exclusion criteria for both groups included a history of secondary headaches (e.g., traumatic brain injury, brain tumors, altered cerebrospinal fluid pressure) based on the 3rd edition of the International Classification of Headache Disorders (ICHD-3) and known psychiatric comorbidities [34,39]. Additional exclusions for MS patients included treatment with interferons, recent relapse, or steroid use within three months prior to recruitment [35,36]. HCs were recruited through community outreach, hospital staff referrals, and volunteer participation, ensuring they had no history of neurological disorders. Eligibility was confirmed through structured interviews and medical record reviews.

### 2.3. Study Procedures and Data Collection

All clinical and imaging measurements were obtained using standard procedures. MS patients underwent a comprehensive clinical assessment from an MS and a headache expert, including a physical and neurological examination to evaluate their Expanded Disability Status Scale (EDSS) score, a widely used and validated measure of disability in MS [40]. The assessment also included a detailed medical history to document previous headache episodes and any other relevant neurological symptoms. A semi-structured headache questionnaire, routinely used at the hospital’s headache specialty clinics, helped identify, diagnose, and categorize headache disorders retrospectively, over the past three months from the day of recruitment, in both the MS group and the HCs based on the ICHD-3 criteria, which are internationally validated for classifying primary headache disorders [36]. The questionnaire was administered by a trained neurologist/headache expert during a face-to-face session, and responses were entered into an electronic database for subsequent analysis. The semi-structured headache questionnaire used for diagnosis is available in the Supplementary Materials. Information regarding headache characteristics such as frequency and duration of episodes, pain location, quality and severity, as well as accompanying symptoms such as nausea, vomiting, photo- or phonophobia, aggravation by routine physical activity, presence of aura or autonomic symptoms, and the impact on daily activities were collected and contributed to headache disorder diagnosis. Additional collected data included demographic details (age, sex, and level of education), as well as disease specifics including MS subtype according to 2017 revised McDonald criteria, a standardized and validated diagnostic framework, age at disease onset and diagnosis, disease duration, disease-modifying treatment, and the most recent relapse. Further clinical variables such as CSF oligoclonal band status and neurological exam findings were drawn from medical records and confirmed through direct evaluation, ensuring both reliability and clinical accuracy. Data were reviewed and cross-checked to reduce measurement error.

### 2.4. Statistical Analysis

Data analysis was performed using StatsDirect statistical software and a significance level of *p* < 0.05 was used for all analyses. A multivariable logistic regression model was used to calculate the odds ratio (OR) for primary headache disorders in MS after adjusting for potential confounders, such as age and sex. In addition, the Mann–Whitney U test was applied to compare EDSS scores between pwMS with and without migraine.

### 2.5. Ethical Considerations

Written informed consent was secured from all participants and the study protocol was approved by the Ethical Committee of Eginition University Hospital (IRB number: 981/22 December 2021). All study procedures were conducted in accordance with the ethical standards of the Helsinki Declaration of 1975, as revised in 2000.

## 3. Results

Ninety-six pwMS and ninety-six matched controls met the inclusion criteria and were included in the study. Among pwMS, 77.1% (*n* = 77) had relapsing–remitting MS (RRMS), 7.3% (*n* = 7) primary progressive MS (PPMS), and 15.6% (*n* = 15) had been diagnosed with secondary progressive MS (SPMS). Baseline demographical and clinical characteristics of both the MS group and the HCs are shown in Table 1.

**Figure 2:**
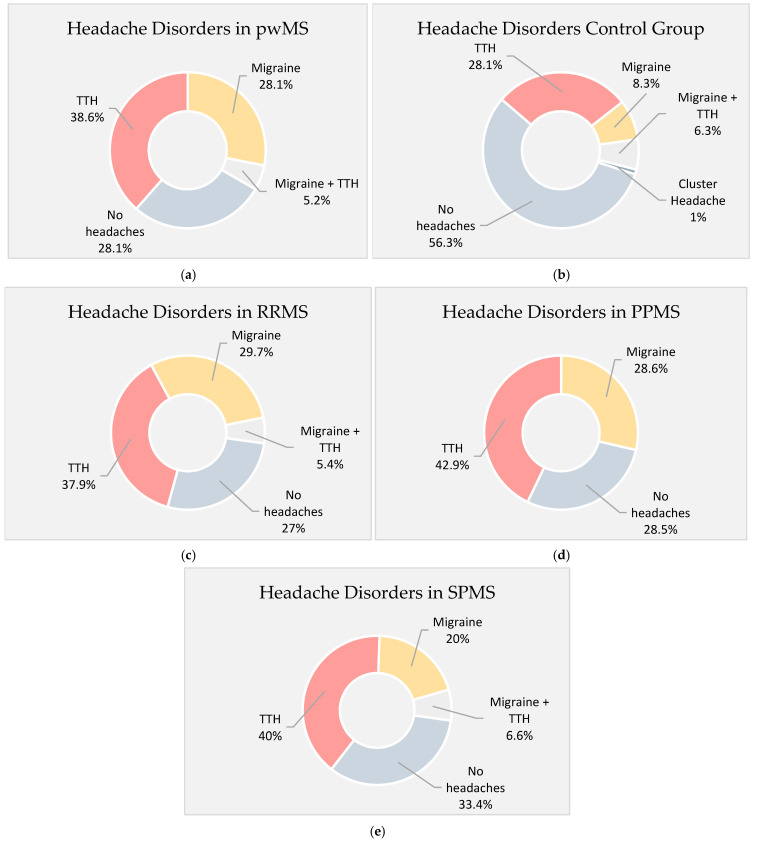
Prevalence of primary headache disorders across study groups. (**a**) People with MS (pwMS); (**b**) control group; (**c**) RRMS; (**d**) PPMS; (**e**) SPMS.

In the MS group, 71.9% of the participants (*n* = 69) were diagnosed with headache. Among them, 28.1% (*n* = 27) fulfilled the criteria for migraine and 38.6% (*n* = 37) for TTH, while 5.2% (*n* = 5) were diagnosed with both conditions. No cases of TACs were diagnosed and 28.1% of the patients did not report any headache at all. Regarding the prevalence of primary headache disorders across different MS subtypes, migraine and TTH were diagnosed in 29.7% and 37.9% of people with RRMS, while 5.2% were diagnosed with both primary headache disorders. Migraine and TTH prevalence in patients with PPMS was 28.6% and 42.9%, respectively, while 28.5% did not experience headaches. Furthermore, migraine was diagnosed in 20% of the patients with SPMS and TTH in 40%, while the diagnosis of both was made in 6.6% of the patients. Finally, in the control group, 8.3% of the participants were diagnosed with migraine, 28.2% of them were diagnosed with TTH, 6.3% were diagnosed with both, and 1% was diagnosed with cluster headache. A total of 43.8% was diagnosed with any primary headache disorder. The prevalence rates of all headache disorders across all study groups are summarized in Figure 2.

Among migraine-affected MS patients (*n* = 32), 12.5% (*n* = 4) had migraine with aura, while 87.5% (*n* = 28) had migraine without aura. Regarding migraine classification, 87.5% (*n* = 28) had episodic migraine, and 12.5% (*n* = 4) had chronic migraine. Headache characteristics for both the MS group and the HCs are summarized in Table 2.

Regarding the association of headache and MS, pwMS were more than three times as likely to be diagnosed with any primary headache disorder (OR = 3.29; 95% CI: 1.80 to 5.99; *p* = 0.0001), more than two times as likely to be diagnosed with migraine (OR = 2.18 95% CI: 1.05 to 4.49; *p* = 0.04), and almost two times as likely to be diagnosed with TTH (OR = 1.82 95% CI: 1.02 to 3.25; *p* = 0.04). After adjusting for age and sex, pwMS were more than four times as likely to be diagnosed with any primary headache disorder (OR = 4.54; 95% CI: 2.28 to 9.04; *p* = 0.17 × 10^−5^) and more than two times as likely to be diagnosed with migraine (OR = 2.21 95% CI: 1.05 to 4.62; *p* = 0.04) or TTH (OR = 2.16 95% CI: 1.16 to 4; *p* = 0.01).

Results are shown in Table 3.

Furthermore, the Mann–Whitney U test comparing EDSS scores between MS patients with and without migraines (*n* = 96) revealed no significant differences (U = 1169.5, *p* = 0.202), indicating that migraine presence does not significantly impact disability levels in this sample.

## 4. Discussion

Our study showed that primary headache disorders are more prevalent and significantly associated with MS in a cohort recruited from the MS outpatient clinic at Eginition University Hospital in Athens, Greece. Nearly 72% of the pwMS were diagnosed with a primary headache disorder, one of the highest rates reported in the literature. More specifically, pwMS were more than twice as likely to experience either migraine or TTH compared to HCs and more than four times as likely to experience any primary headache disorder. Migraine was diagnosed in 28.1% and TTH in 38.5% of the patients. No cases of cluster headache or other trigeminal autonomic cephalalgias were observed among pwMS compared to one case identified in the control group. This outcome is consistent with epidemiological data, as these headache types are known to have a very low prevalence in the general population [34]. Historically, the association between migraine and MS is well known and has been demonstrated in several case–control studies and meta-analyses [28,29]. In contrast, TTH has not been previously associated with MS; in fact, our findings show a higher prevalence of TTH in pwMS compared to the general population. To our knowledge, our study is the first to report such a high prevalence and association between TTH and MS.

Comparatively, our findings show a prevalence of migraine in pwMS (28.1%) that aligns closely with the reported prevalence in the literature, where two meta-analyses indicate an approximate prevalence of 30% [28,29]. This similarity suggests a consistent pattern of migraine occurrence among MS patients across different studies. However, our results for TTH indicate a higher prevalence (38.5%) compared to the 19% typically noted in the literature, as reported by the same meta-analysis [28]. The notably higher prevalence of TTH in our study may reflect limitations in previous methodologies or unique demographic or clinical characteristics of our study population that predispose them to higher rates of TTH. This divergence underscores the need for further research into the factors contributing to the increased TTH prevalence among pwMS. Additionally, the lack of cluster headaches and other TACs in our findings is consistent with their documented low occurrence in MS populations and the general population. In terms of disease subtypes, there were no significant differences in the prevalence of primary headache disorders among different MS phenotypes, except in patients with SPMS, where the prevalence of migraine was lower (20%), but still significantly higher compared to the control group. This observation could be partly explained by age-related mechanisms, since it is known that the frequency and intensity of migraines tend to decrease with age and SPMS is typically diagnosed in older adults who have transitioned from RRMS to SPMS. Additionally, it could be speculated that the increased permeability of the blood–brain barrier and consequent lymphocyte trafficking from the periphery to the CNS during the relapsing stages of the disease might be key drivers of the increased prevalence of migraine. Conversely, the compartmentalized inflammation and the increased involvement of innate immunity, such as microglia, in the progressive stages of the disease might not trigger the pathophysiological cascades associated with migraine as significantly, a speculation that warrants further research.

Several cross-sectional and case–control studies have attempted to investigate the relationship between primary headache disorders and MS, but results have been contradictory, mostly due to methodological weaknesses such as the absence of control groups, small sample sizes, and the lack of headache diaries, which would assist in the diagnosis of primary headache disorders [5,6,7,8,9,10,11,12,13,14,15,16,17,18,19,20,21,22,23,24,25,26,27]. In our study, these factors were addressed. More specifically, a power calculation was performed to determine the required sample size, and a control group of healthy individuals was recruited for comparison with pwMS. Additionally, a semi-structured headache questionnaire, utilized at the headache specialty clinics of our hospital, was employed to diagnose and categorize headache disorders in both the MS group and the healthy controls based on the 3rd edition of the International Classification of Headache Disorders (ICHD-3) criteria.

Although it appears that pwMS are more likely to experience primary headache disorders, the underlying mechanisms of this association remain elusive. Various theories have been proposed, though none are universally accepted. A leading theory suggests that demyelinating lesions in areas of the brain responsible for pain modulation and perception, such as the brainstem nuclei including the periaqueductal gray (PAG), play a significant role. The PAG is crucial in pain modulation by inhibiting pain signal transmission at the brainstem level, which decreases central sensitization and reduces nociceptive input from the periphery to the CNS. Dysfunction of the PAG, due to demyelination and subsequent neurodegeneration, can lead to a lowered pain threshold and heightened sensitivity to noxious stimuli. Additionally, dysfunction of other critical structures for pain modulation and perception, such as the thalamus or cortex, may increase pain perception and contribute to the development of primary headache disorders. Another theory focuses on immunological changes in lymphocyte populations in pwMS. This theory suggests that the presence of activated lymphocytes in the CNS of pwMS is linked to increased expression of calcitonin gene-related peptide (CGRP), a key molecule in migraine pathogenesis. Consequently, pwMS may be more susceptible to the effects of CGRP, leading to a higher frequency of migraines. However, this cannot explain the higher prevalence of TTH, as the role of CGRP in TTH is less clear. The increased prevalence of migraine and ΤΤH in pwMS has significant implications for clinical practice. It is important to identify and treat these patients promptly as headaches can severely affect their quality of life. Using targeted questions to assess the frequency and characteristics of headaches is crucial for guiding treatment decisions. For those with a history of headaches, routine use of headache diaries can help classify the headache type and dictate treatment strategies. The management of headaches in pwMS follows the same principles as in non-MS individuals and includes both acute and prophylactic treatment, the latter being for those with frequent or disabling attacks. However, caution is needed regarding drug-to-drug interactions and the potential adverse effects of medications. Specific preventive medications, such as antiepileptics and tricyclic antidepressants, should be carefully prescribed due to their potential to impair cognition and deteriorate quality of life [41,42,43]. Duloxetine and venlafaxine can effectively contribute to the reduction in monthly headache days, while duloxetine can be effective in managing sensory symptoms as well [44]. Another well-tolerated option is candesartan due to its favorable safety profile [45]. Limited experience exists on the use of anti-CGRP monoclonal antibodies; however, there is increasing data supporting their use in pwMS [4]. Treatment options should be thoroughly discussed with patients, ensuring decisions are tailored to their individual characteristics and needs. A holistic approach, including lifestyle modifications such as smoking cessation, limited alcohol consumption, a well-balanced diet, adequate sleep, and the management of comorbid conditions such as anxiety and depression, is essential for enhancing treatment outcomes.

Finally, several limitations of this study should be acknowledged. First of all, this is a case–control study in which pwMS and controls were asked about the occurrence and frequency of headaches in retrospect. This could result in recall bias, particularly in pwMS who are possibly cognitively impaired. Additionally, since all participants were recruited from a single center, there is a risk of selection bias. Furthermore, our study assesses the association between MS and headache disorders using logistic regression; however, it does not investigate potential biological mechanisms driving this relationship. Additionally, due to the retrospective design, we cannot establish a temporal or causal link between MS and headache. Future studies incorporating longitudinal designs and mechanistic analyses are needed to further explore the factors contributing to this association.

## 5. Conclusions

In conclusion, migraine and TTH are associated with MS and are both more than twice as frequent in pwMS compared to healthy controls in a cohort recruited from the MS outpatient clinic at Eginition University Hospital in Athens, Greece. Prospective, longitudinal follow-up of these groups is essential to establish robust conclusions about the potential association and prevalence of primary headache disorders in pwMS.

## Figures and Tables

**Table 1:** Participants’ demographics and clinical data.

Demographical and Clinical Data		
	MS	Controls
Gender		
Female—*n* (%)	69 (71.9)	66 (68.75%)
Male—*n* (%)	27 (28.1)	30 (31.25%)
Ethnicity—*n* (%)		
Caucasian	96 (100)	96 (100)
Age (years) mean ± SD	42.15 (12.81)	37.16 (12.63)
Presenting symptom for pwMS—*n* (%)		
Sensory	33 (34%)	
Optic neuritis	19 (20%)	
Motor	14 (15%)	
Balance	8 (8%)	
Diplopia	7 (7%)	
Polysymptomatic	12 (13%)	
Bladder symptoms	2 (2%)	
Pain	1 (1%)	
Diagnosis Method		
Imaging + LP	88 (92%)	
Imaging only	8 (8%)	
Exclusion of Other Diagnoses		
Imaging + CSF analysis + blood tests (MOG, AQP4)	62 (65%)	
Imaging + CSF analysis	26 (27%)	
Imaging only	8 (8%)	
Mean age at MS diagnosis ± SD	32.5 (6.3)	
Disease duration (years) mean ± SD	8.57 (8.52)	
EDSS mean ± SD	3.13 (1.88)	
Disease subtype		
RRMS—*n* (%)	77 (77.1)	
PPMS—*n* (%)	7 (7.3)	
SPMS—*n* (%)	15 (15.6)	
LP findings		
Oligoclonal bands positive	78 (81%)	
Oligoclonal bands negative	10 (10%)	
Did not undergo LP	8 (8%)	
Imaging Findings		
Mean number of total MS lesions ± SD	15.8 ± 6.3	
Active lesions ± SD	0.24 ± 0.67	
Black holes ± SD	3.26 ± 0.69	
Disease modifying treatments—*n* (%)		
Ocrelizumab	41 (43.16)	
Dimethyl-Fumarate	13 (13.68)	
Glatiramer Acetate	10 (10.53)	
Natalizumab	7 (7.37)	
None	7 (7.37)	
Fingolimod	6 (6.32)	
Rituximab	3 (3.16)	
Teriflunomide	3 (3.16)	
Interferon beta-1a	2 (2.11)	
Ofatumumab	1 (1.05)	
Mycophenolate Mofetil	1 (1.05)	

*n*: number of patients; SD: standard deviation; MS: multiple sclerosis; RRMS: relapsing–remitting multiple sclerosis; SPMS: secondary progressive multiple sclerosis; PPMS: primary progressive multiple sclerosis; EDSS: Expanded Disability Status Scale; LP: lumbar puncture; CSF: cerebrospinal fluid; MOG: myelin oligodendrocyte glycoprotein; AQP4: aquaporin 4.

**Table 2:** Headache characteristics in pwMS and HCs.

Headache Characteristics		
	MS	Controls
Migraine—*n* (%)	32 (33.3)	14 (14.5)
Episodic—*n* (%)	28 (87.5)	12 (85.7)
Chronic—*n* (%)	4 (12.5)	2 (14.3)
Aura—*n* (%)	4 (12.5)	1 (7.1)
Photo/phonophobia—*n* (%)	21 (77%)	10 (71.4%)
Mean pain intensity (VAS ± SD)	7.3 ± 1	6.4 ± 1.3
Mean episode duration (hours) ± SD)	17 ± 20	17 ± 20
Mean headache days per month ± SD	5.5 ± 5.4	2.6 ± 1.5
TTH—*n* (%)	42 (43.7)	33 (34.6)
FTTH—*n* (%)	39 (93%)	30 (90.9)
CTTH—*n* (%)	3 (7%)	3 (9.1)
Mean pain intensity (VAS)± SD)	5.7 ± 1.3	5.3 ± 1.3
Mean episode duration (hours) ± SD	5.5 ± 9.3	2.2 ± 1.1
Mean headache days per month ± SD	3.4 ± 4.7	2.7 ± 2.7

*n*: number of patients; SD: standard deviation; VAS: visual analog scale; TTH: tension-type headache; FTTT: frequent tension-type headache; CTTH: chronic tension-type headache.

**Table 3:** Association between primary headache disorders and MS.

	Unadjusted		Adjusted	
	OR (95% CI)	*p*-Value	OR (95% CI)	*p*-Value
Any headache	3.29 (1.80 to 5.99)	*p* = 0.0001	4.54 (2.28 to 9.04)	*p* = 0.17 × 10^−5^
Migraine	2.18 (1.05 to 4.49)	*p* = 0.04	2.21 (1.05 to 4.63)	*p* = 0.04
TTH	1.82 (1.02 to 3.25)	*p* = 0.04	2.16 (1.16 to 4)	*p* = 0.01

MS: multiple sclerosis; TTH: tension-type headache.

## Data Availability

Dataset available on request from the authors.

## Supplementary Materials

The following supporting information can be downloaded at https://www.mdpi.com/article/10.3390/jcm14082778/s1, Supplementary Material S1—STROBE checklist. Supplementary Material S2—Headache Diary.

## Abbreviations

The following abbreviations are used in this manuscript:

CGRP	Calcitonin Gene-Related Peptide
CNS	Central Nervous System
CSF	Cerebrospinal Fluid
DRN	Dorsal Raphe Nucleus
EDSS	Expanded Disability Status Scale
HC	Healthy Controls
ICHD-3	International Classification of Headache Disorders, 3rd Edition
LC	Locus Coeruleus
MS	Multiple Sclerosis
OR	Odds Ratio
PAG	Periaqueductal Gray
PPMS	Primary Progressive Multiple Sclerosis
pwMS	People with Multiple Sclerosis
RRMS	Relapsing-Remitting Multiple Sclerosis
SD	Standard Deviation
SPMS	Secondary Progressive Multiple Sclerosis
TTH	Tension-Type Headache
