# Supplementary material for: Migraine and Tension-Type Headache Are Associated with Multiple Sclerosis: A Case–Control Study
Source: J Clin Med. 2025 Apr 17;14(8):2778. doi: 10.3390/jcm14082778 (PMC12027967; doi:10.3390/jcm14082778)
Supplement: Supplementary file 1 [file jcm-14-02778-s001.zip › STROBE checklist.pdf]

STROBE Statement—checklist of items that should be included in reports of observational studies

	Item No	Recommendation	Where It Is Addressed (Line Numbers)
**Title and abstract**	1	(*a*) Indicate the study's design with a commonly used term in the title or the abstract	Lines 2-3
		(*b*) Provide in the abstract an informative and balanced summary of what was done and what was found	Lines 14-40
**Introduction**			
Background/rationale	2	Explain the scientific background and rationale for the investigation being reported	Lines 45-122
Objectives	3	State specific objectives, including any prespecified hypotheses	Lines 123-133
**Methods**			
Study design	4	Present key elements of study design early in the paper	Lines 136-151
Setting	5	Describe the setting, locations, and relevant dates, including periods of recruitment, exposure, follow-up, and data collection	Lines 149-151
Participants	6	(*a*) *Cohort study*—Give the eligibility criteria, and the sources and methods of selection of participants. Describe methods of follow-up<br>*Case-control study*—Give the eligibility criteria, and the sources and methods of case ascertainment and control selection. Give the rationale for the choice of cases and controls<br>*Cross-sectional study*—Give the eligibility criteria, and the sources and methods of selection of participants	Lines 154 - 167
		(*b*) *Cohort study*—For matched studies, give matching criteria and number of exposed and unexposed<br>*Case-control study*—For matched studies, give matching criteria and the number of controls per case	
Variables	7	Clearly define all outcomes, exposures, predictors, potential confounders, and effect modifiers. Give diagnostic criteria, if applicable	Lines 170-195
Data sources/ measurement	8*	For each variable of interest, give sources of data and details of methods of assessment (measurement). Describe comparability of assessment methods if there is more than one group	Lines 170-195
Bias	9	Describe any efforts to address potential sources of bias	Lines 154-167
Study size	10	Explain how the study size was arrived at	Lines 141-148
Quantitative variables	11	Explain how quantitative variables were handled in the analyses. If applicable, describe which groupings were chosen and why	Lines 170-195

Statistical methods	12	(*a*) Describe all statistical methods, including those used to control for confounding	Lines 198-202
		(*b*) Describe any methods used to examine subgroups and interactions	Lines 198-202
		(*c*) Explain how missing data were addressed	NA
		(*d*) *Cohort study*—If applicable, explain how loss to follow-up was addressed<br>*Case-control study*—If applicable, explain how matching of cases and controls was addressed<br>*Cross-sectional study*—If applicable, describe analytical methods taking account of sampling strategy	NA
		(*e*) Describe any sensitivity analyses	NA

Continued on next page

Results			Where It Is Addressed (Line Numbers)
Participants	13*	(a) Report numbers of individuals at each stage of study—eg numbers potentially eligible, examined for eligibility, confirmed eligible, included in the study, completing follow-up, and analysed	Lines 210-214 and
		(b) Give reasons for non-participation at each stage	NA
		(c) Consider use of a flow diagram	NA
Descriptive data	14*	(a) Give characteristics of study participants (eg demographic, clinical, social) and information on exposures and potential confounders	Lines 210-214 & Table 1
		(b) Indicate number of participants with missing data for each variable of interest	NA
		(c) *Cohort study*—Summarise follow-up time (eg, average and total amount)	NA
Outcome data	15*	*Cohort study*—Report numbers of outcome events or summary measures over time	*NA*
		Case-control study—Report numbers in each exposure category, or summary measures of exposure	*Lines 223-236 and 270-277*
		Cross-sectional study—Report numbers of outcome events or summary measures	*Lines 223-236*
Main results	16	(*a*) Give unadjusted estimates and, if applicable, confounder-adjusted estimates and their precision (eg, 95% confidence interval). Make clear which confounders were adjusted for and why they were included	Lines 270-277
		(*b*) Report category boundaries when continuous variables were categorized	NA
		(*c*) If relevant, consider translating estimates of relative risk into absolute risk for a meaningful time period	NA
Other analyses	17	Report other analyses done—eg analyses of subgroups and interactions, and sensitivity analyses	Lines 281-284
**Discussion**			
Key results	18	Summarise key results with reference to study objectives	Lines 286-293
Limitations	19	Discuss limitations of the study, taking into account sources of potential bias or imprecision. Discuss both direction and magnitude of any potential bias	Lines 378-387
Interpretation	20	Give a cautious overall interpretation of results considering objectives, limitations, multiplicity of analyses, results from similar studies, and other relevant evidence	Lines 339-377
Generalisability	21	Discuss the generalisability (external validity) of the study results	Lines 389-393
**Other information**			
Funding	22	Give the source of funding and the role of the funders for the present study and, if applicable, for the original study on which the present article is based	NA

*Give information separately for cases and controls in case-control studies and, if applicable, for exposed and unexposed groups in cohort and cross-sectional studies.

Note: An Explanation and Elaboration article discusses each checklist item and gives methodological background and published examples of transparent reporting. The STROBE checklist is best used in conjunction with this article (freely available on the Web sites of PLoS Medicine at http://www.plosmedicine.org/, Annals of Internal Medicine at http://www.annals.org/, and Epidemiology at http://www.epidem.com/). Information on the STROBE Initiative is available at www.strobe-statement.org.
