# Supplementary figures and images for: Migraine and Tension-Type Headache Are Associated with Multiple Sclerosis: A Case–Control Study
Source: J Clin Med. 2025 Apr 17;14(8):2778. doi: 10.3390/jcm14082778 (PMC12027967; doi:10.3390/jcm14082778)

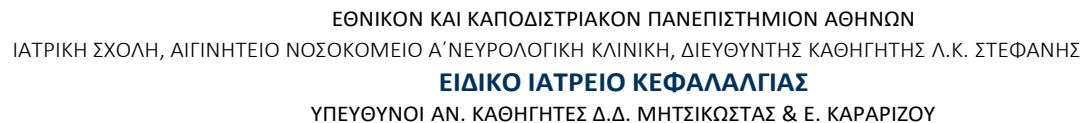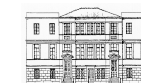

Τηλ. 2107251315

FULL NAME \_\_\_\_\_

[illegible]

Supplement: Supplementary file 1 [file jcm-14-02778-s001.zip › Headache-diary-ENG.pdf]
